# Evaluation of an isothermal amplification HPV detection assay for primary cervical cancer screening

**DOI:** 10.1186/s13027-020-00328-1

**Published:** 2020-10-23

**Authors:** Wei Zhang, Hui Du, Xia Huang, Chun Wang, Xianzhi Duan, Yan Liu, Bin Shi, Wei Zhang, Xinfeng Qu, Lihui Wei, M. Schiffman, J. L. Belinson, Ruifang Wu

**Affiliations:** 1 Peking University Shenzhen Hospital, Shenzhen, 518036 P. R. China; 2 Shenzhen Key Laboratory on Technology for Early Diagnosis of Major Gynecological diseases, Shenzhen, P. R. China; 3 Capital Medical University Beijing Tongren Hospital, Beijing, P. R. China; 4 Fudan University Huanshan Hospital, Shanghai, P. R. China; 5 The second Hospital of Hebei Medical University, Shijiazhuang, P. R. China; 6 Wuhan University Zhongnan Hospital, Wuhan, P. R. China; 7 Expert in Three Engineering Office, Shenzhen Maternal of Peking University Shenzhen Hospital, Shenzhen, 518036 China; 8 Department of Obstetrics and Gynecology, Peking University People’s Hospital, Beijing, P. R. China; 9 National Cancer Institute, Division of Epidemiology and Genetics, Bethesda, USA; 10 Women’s Health Institute, Cleveland Clinic, Cleveland, OH USA; 11 PPreventive Oncology International, Inc., Shaker Heights, USA

**Keywords:** AmpFire assay, Human papillomavirus, Self-collection, Clinician-collection, Isothermal amplification

## Abstract

**Objective:**

The aim of this research was to evaluate independently the performance of a new isothermal amplification assay for cervical cancer screening compared to two previously validated PCR-based assays and histologic endpoints.

**Methods:**

This is a sub-study from the Chinese multi-center screening trial (CHIMUST). The self-collected and clinician-collected specimens stored in PreservCyt at − 4 °C from 6042 women with complete data were tested with the AmpFire assay. These specimens had been previously tested with Cobas and SeqHPV assays. In the primary study all patients with an abnormal test were referred to colposcopy where all had directed and/or random biopsies plus ECC. No additional patients were called back based on the AmpFire results.

**Results:**

6042/6619 women had complete data (mean age 44.1). There were 57 cases of CIN 2, 35 cases of CIN 3 and 2 cancers. The sensitivity for CIN2+ and CIN3+ were similar among the three assays (both direct and self-collected). For the specificities in all categories (CIN2+/CIN3+ and self and direct collection), isothermal amplification assay was either equal to or more specific than Cobas but consistently less specific than SeqHPV.

**Conclusion:**

The AmpFire HPV assay showed similar sensitivity to Cobas and SeqHPV for CIN2+ and CIN3+ on both self and clinician-collections (*P*>0.05), with good specificity. The speed, low cost, and simplicity of this assay will make it particularly suited for low and middle resource settings. Its accuracy with self-collection makes it applicable for mass screening programs.

## Introduction

For over two decades, our academic research group has had a major interest in self-collection technologies for cervical cancer screening [1]. We initially demonstrated how, depending on the assay used, a self-collected vaginal specimen could achieve sensitivity equal to a direct endocervical specimen (clinician-collected) [2]. We have concentrated on lower-resource settings, and initially evaluated faster assay platforms that could accommodate the volume of samples that could be delivered with self-collection [3, 4]. Then we developed a solid media transport card that would be affordable in medically underserved areas to avoid the handling and transport of liquid media [5]. Also, we developed community-based healthcare delivery systems and developed approaches that could reach thousands of women per day [6, 7].

Recently, we learned of the AmpFire assay which presented a unique constellation of characteristics that appeared special among the sea of high-risk human papillomavirus (hrHPV) assays we had explored over the years. The amplification method used was isothermal, making it simple and fast compared to standard thermocycling PCR (polymerase chain reaction). With the simplicity came a significant reduction in per sample cost, as well as the equipment and space required, equal to the least expensive of HPV assays. In addition, there was no deoxyribonucleic acid (DNA) extraction required, since the assay handles raw sample. Therefore, we saw a future opportunity to use a simple dry collection brush with no transport media required (solid or liquid). This would further reduce the cost and facilitate population-based screening in remote communities. The combination of these unique features also represented a real opportunity to integrate clinic-based hrHPV testing into same-day “test-and-treat” cervical screening programs. The platform offers multiplex detection in a single tube of 15 high-risk types with separate detection of types 16/18; or optionally offers full genotyping in 4 tubes. Also, using the same equipment and similar isothermal methodology a panel of STD tests could be done, including SARS-CoV-2 (https://atilabiosystems.com/our-products/). When we first became aware of the AmpFire technology, there were promising analytic data, but we were unable to find clinical validation against histologic outcomes [8]. Therefore, the objective of this study was to compare the AmpFire high-risk HPV assay to two previously validated PCR-based assays, using histologic endpoints.

## Methods

### Study population

The Chinese Multicenter Screening Trial (CHIMUST) “screening phase” took place between August 2016 and November 2017 [9]. The study was IRB approved by Peking University Shenzhen Hospital (PUSHGYN 2015005) and registered with the WHO designated Chinese Clinical Trial Registry (ChiCTR-EOC-16008456). CHIMUST was designed to compare the standard PCR based assay (Cobas assay, Roche, Pleasanton CA, USA) and a next-gen sequencing assay (SeqHPV assay, BGI Shenzhen, PRC) on both self- and clinician direct collected specimens. It was also designed to study liquid vs solid media sample transport for self-collected specimens. This sub-study of the isothermal amplification assay (AmpFire assay) focuses on 6619 patients from several of the study locations, whose samples were stored at − 4 °C and not frozen [Hebei Province (Pingxiang) – 2035; Hubei Province (Huang Shi) – 1250; Guangdong Province (Chao Zhou) – 1000; Beijing (Men Tao Go) – 988; Inner Mongolia (Xiang Huang Qi) – 1346]. Women aged 30–55 years who had not been screened for cervical cancer in the 3 years prior to entering the study were enrolled. Eligibility required they be non-pregnant, have an intact uterus, and have had no prior pelvic radiation. All the women signed an informed consent for the study, and for the storage and future use of their specimens. The protocol of this trial was approved by the ethics committee of Peking University Shenzhen Hospital (IRB: PUSHGYN 2016001).

### Specimen collection

In the primary study (CHIMUST) every woman first took a self-sample, rubbed the brush (with standard 9- μm nylon “Christmas tree head) on the POI solid media transport card [5] and then placed the brush in 6 ml of ThinPrep medium (PreservCyt). Next a speculum was placed, and a physician obtained a direct endocervical specimen also placed in PreservCyt medium (20 cc). Self-collection was processed with the Cobas PCR-based assay and next-gen sequencing assay. The clinician collection was processed for the same two assays, and also for cytology (ThinPrep, Hologic, Marlborough, MA, USA). Some of the patient’s samples in CHIMUST were stored as frozen specimens. There were 6619 patients whose samples (both self and direct collected) in ThinPrep were stored at − 4 °C. These were the specimens tested with the isothermal amplification assay for this study. The specimens had been stored from 1 to 3 years depending on the entry time of the patients into the study at the various study sites.

### Isothermal amplification assay (AmpFire HPV assay)

AmpFire is an isothermal amplification real-time fluorescent HPV detection assay, developed in the USA (Atila BioSystems, Inc., Mountain View, CA), which detects HPV directly from clinical samples. No DNA extraction step is required [8] (https://atilabiosystems.com/multiplex-high-risk-hpv-by-fluorescent-detection/). Using a dry brush sample or the isothermal amplification assay transport media total processing time is ≤ 1 h. Using ThinPrep samples requires initial removal of the methanol. There are two isothermal amplification platforms, a multiplex assay: 16, 18, with a thirteen-type high risk pool; and a genotyping assay for 15 specific types. The platforms can be run individually, simultaneously or sequentially. Samples can be processed individually or batched. Formalin fixed paraffin embedded tissue blocks (FFPE) are also acceptable for genotyping studies. The multiplex assay detects 15 “high risk” HPV types: 16, 18, 31, 33, 35, 39, 45, 51, 52, 53, 56, 58, 59, 66, and 68 in a single tube reaction and simultaneously identifies specifically the presence of types 16 and 18 with real time fluorescent detection [10–12]. The human cellular gene beta-globin is used as an internal control to measure sample adequacy. Both the isothermal amplification multiplex assay and genotyping assay were Conformité Européenne (European Community) CE-marked in 2017 and received Chinese Food and Drug Administration CFDA) approval in December 2015.

### The standard PCR based assay HPV assay (COBAS HPV assay)

The Cobas 4800 HPV Test was Conformité Européenne (European Community) CE-marked in 2009 and received US Food and Drug Administration approval in April 2011. The Cobas 4800 system platform (Roche Molecular Diagnostics, Pleasanton, CA.), consists of the Cobas × 480 instrument and the Cobas z480 analyzer. It features fully automated nucleic acid extraction in combination with real-time PCR technology plus software that integrates the two components respectively.HPV 16 and 18 are identified separately while 12 other HR-HPV types (31, 33, 35, 39, 45, 51, 52, 56, 58, 59, 66 and 68) are detected as a pool. The human cellular gene beta-globin is used as an internal control to measure the sample adequacy and the quality of extraction and amplification.The test was performed according to the manufacturer’s instructions (https://www.hpv16and18.com/labs/lab-efficiencies/COBAS-4800-system.html) [13].

### SeqHPV assay

The SeqHPV assay is based on next-generation genomic sequencing. The technology employs a series of unique primers to amplify about 150 base pairs DNA of the L1 gene, and a pair of primers to amplify about 150 base pairs of the human β-globin gene as the internal quality control for identifying the false negatives caused by inadequate DNA or failed PCR. The assay uses multiplex PCR to individually detect a total of 14 HR-HPV genotypes (16, 18, 31, 33, 35, 39, 45, 51, 52, 56, 58, 59, 66, 68). The assay is approved by the CFDA [3, 4].

### Colposcopy and histology examination

Women who tested positive by either the Cobas PCR based assay or next gen sequencing assay on self-collected or clinician-collected specimens, or who had cytology ≥ ASC-US were asked to return for colposcopy. Women having colposcopy were evaluated by our standard research protocol by having a minimum of four small cervical biopsies plus an endocervical curettage [Preventive Oncology International, Inc. (POI) protocol of directed and random biopsies by quadrant] [1]. Histology results from all study sites were independently reassessed by a study gyn pathologist. All histology slides were interpreted as Normal, CIN 1, CIN 2, CIN 3/AIS, or cancer. No additional patients were recalled for a colposcopic examination based on the results of the isothermal amplification assay.

### Statistical analysis

HPV type 53 (included only in the isothermal amplification assay) was eliminated from the analysis so a 14- type comparison could be done among the three assays.

Differences in sensitivity and specificity for ≥CIN2,3 were compared using McNemar’s test. *P* < 0.05 was considered significant. All data were analyzed by SPSS 19.0 [14].

### Role of the funding source

This work was supported by a grant from the Shenzhen Health Family Planning Commission, Shenzhen, PR China (Sanming Project of Medicine in Shenzhen, Protocol Number SZSM201412010) and the governmental funds for Shenzhen Leading Gynecological Subject, Shenzhen, PR China,(Science and Education of Shenzhen Health, Protocol Number [2018]61). The two funders had no role whatsoever in the study conduct or analysis.

## Results

A total of 6042 women who had all prescribed screening and diagnostic procedures with no missing data are included in the analysis. The mean age of the study population (6042) was 44.1 years. 577 (8.7%) women were dropped from the analysis: 1501 women were asked to return for colposcopy, and 556 (37.04%, 556/1501) did not return to the study doctors. 6 (0.09%, 6/6619) were missing Cobas HPV clinician collection data; 1 (0.02%, 1/6619) was missing SeqHPV clinician data; 10 (0.15%, 10/6619) were missing SeqHPV self-collection results (1 of the these also missing AmpFire self-collection); and 4 (0.06%, 4/6619) had unsatisfactory cytology. The 577 women who were excluded were similar to the total dataset of 6042. 942 women underwent colposcopy and biopsies. Considered as an “intention to treat” analysis, CIN 2 was diagnosed in 0.94% (57/6042) of the total screened population in the analysis, CIN 3 in 0.58% (35/6042) (one of the CIN3 also had AIS), cervical cancer (squamous cell carcinoma) in 0.03% (2/6042). The comparable risk percentages had all women followed protocol, presuming the exclusions were random, would be approximately 1.6 times higher.

The positivity rates of the three assays, and two collection methods, were very similar. Neither assay choice nor clinician versus self-sampling led to important differences in HPV positivity: The positivity rate of the AmpFire HPV assay for clinician collection and self-collection were 10.6% (639/6042) and 11.5% (697/6042), respectively. The positivity rates of Cobas HPV for clinician collection and self-collection were 10.3% (620/6042) and 12.8% (775/6042), respectively. The positivity rates of SeqHPV clinician collection and self-collection were 10.0% (605/6042) and 10.6% (641/6042), respectively. In contrast, the total abnormal cytology (ASC-US or worse) rate was lower than HPV testing, 4.6% (279/6042), with frequencies of ASC-US of 1.9% (114/6042), LSIL of 1.5% (89/6042), ASC-H of 0.45% (27/6042), HSIL of 0.81% (49/6042).

Tables 1 and 2 show the sensitivity and specificity for the three HPV assays for both clinician-collected and self-collected specimens as well as liquid-based cytology.

**Table 1:** Comparison of the sensitivity and specificity for ≥CIN2 of physician-collected specimens (endocervical), and vaginal self-collected assayed for HR-HPV by AmpFire, Cobas, SeqHPV (95% CIs and the actual patient numbers are in parentheses)

Specimen/High-Risk HPV Test	Sensitivity for ≥ CIN 2 (%) (CI) (n)	Specificity for ≥ CIN 2 (%) (CI) (n)
Clinician Collected / AmpFire	95.74% (88.85–98.63) (90/94)	90.77% (90.00–91.49) (5399/5948)
Self-Collected / AmpFire	96.81% (90.29–99.17) (91/94)	89.81% (89.01–90.56) (5342/5948)
Clinician Collected / Cobas	92.55% (84.75–96.70) (87/94)	91.04% (90.28–91.75) (5415/5948)
Self-Collected / Cobas	95.74% (88.85–98.63) (90/94)	88.48% (87.64–89.28) (5263/5948)
Clinician Collected / SeqHPV	91.49% (83.44–95.99) (86/94)	91.27% (90.52–91.97) (5429/5948)
Self-collected / SeqHPV	94.68% (87.45–98.03) (89/94)	90.72% (89.95–90.15) (5396/5948)
Cytology	72.34% (62.00–80.83) (68/94)	96.45% (95.94–96.90%) (5737/5948)

McNemar’s p-value for the comparison of the sensitivity of direct endocervical collected AmpFire to direct endocervical collected Cobas is 0.37 and direct collected SeqHPV is 0.22. McNemar’s p-value for the comparison of the sensitivity of self-collected AmpFire to self-collected Cobas is 1 and self-collected SeqHPV is 0.68. McNemar’s *p*-value for the comparison for the specificities of direct endocervical collected AmpFire to direct endocervical collected Cobas is 0.12 and direct endocervical collected SeqHPV is <0.05. McNemar’s p-value for the comparison for the specificities of self-collected AmpFire to self-collected Cobas and SeqHPV both are <0.05. The HPV tests details of the three CIN2+ missed on self-collected AmpFire are as follows: 1. 33 year female, positive only on direct endocervical collected Cobas with normal cytology; 2. 39 year female, positive on direct endocervical and self-collected Cobas and SeqHPV with LSIL cytology; 3. 54 year female, positive only on self-collected SeqHPV with normal cytology

**Table 2:** Comparison of the sensitivity and specificity for ≥CIN3 of physician-collected specimens (endocervical), and vaginal self-collected assayed for HR-HPV by AmpFire, Cobas, SeqHPV (95% CIs and the actual patient numbers are in parentheses)

Specimen/High-Risk HPV Test	Sensitivity for ≥ CIN 3 (%) (CI) (n)	Specificity for ≥ CIN 3 (%) (CI)(n)
Clinician Collected / AmpFire	100% (88.29–100) (37/37)	89.98% (89.18–90.72) (5403/6005)
Self-Collected / AmpFire	100% (88.29–100) (37/37)	89.01% (88.18–89.78) (5345/6005)
Clinician Collected / Cobas	100% (88.29–100) (37/37)	90.29% (89.51–91.02) (5393/6005)
Self-Collected / Cobas	97.30% (84.19–99.86) (36/37)	87.70% (86.83–88.51) (5266/6005)
Clinician Collected / SeqHPV	100% (88.29–100) (37/37)	90.54% (89.77–91.26) (5393/6005)
Self-collected / SeqHPV	100% (88.29–100) (37/37)	89.94% (89.15–90.69) (5393/6005)
Cytology	100% (88.29–100) (37/37)	95.97% (95.43–96.45%) (5763/6005)

Using McNemar’s for the comparison of the sensitivity of direct endocervical collected AmpFire (100.0%) to direct endocervical collected Cobas (100.0%) and SeqHPV (100.0%) both are *p* = 1.0; the sensitivity of self-collected AmpFire (100.0%) to self-collected Cobas (97.3%) and SeqHPV (100.0%) both are also *p* = 1.0. Using McNemar’s for the comparison of the specificities of direct endocervical collected AmpFire (90.0%) to direct endocervical collected Cobas (90.3%) is *p* = 0.09 and direct endocervical collected SeqHPV (90.5%) is *p* = 0.007; the comparison for the specificities of self-collected AmpFire (89.0%) to self-collected Cobas (87.7%) and SeqHPV (89.9%) both are *p* = <0.001

For ≥CIN2, the sensitivities of the 3 HPV assays for both self and clinician collection were similar. With regard to specificity, for clinician collection, AmpFire was similar to Cobas but less specific than SeqHPV (*p* < 0.05). For self-collection: Cobas was less specific than AmpFire (p < 0.05), and AmpFire less specific than SeqHPV (p < 0.05) (Table 1).

For ≥CIN3, the sensitivities of the 3 HPV assays for both self and direct collection were similar (*p* = 1.0). Regarding specificity, for direct collection AmpFire was similar to COBAS (*p* = 0.09) but less specific than SeqHPV (*p* = 0.007). For self-collection: Cobas was less specific than AmpFire (*p* < 0.001), and AmpFire less specific than SeqHPV (*p* < 0.001) (Table 2).

Table 3 provides a comparison of AmpFire assay to Cobas for type detection; agreement was very good to excellent based on the kappa statistic. The histologic outcomes for the discrepant results were examined, and did not reveal important differences; specifically, there was only 1 case of ≥CIN3 with discrepant typing results, of the many thousands of typing results.

**Table 3:**
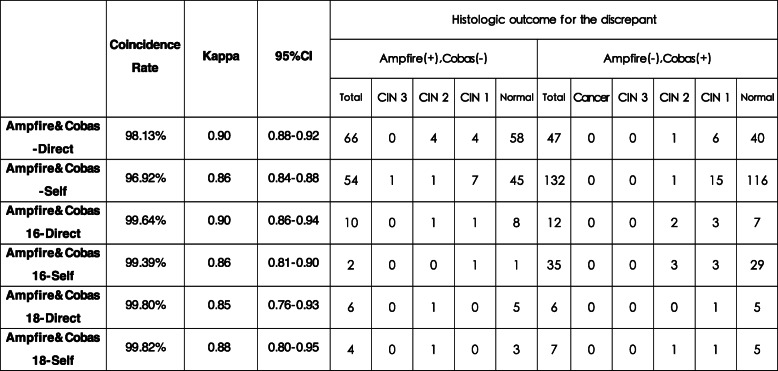
Comparison of the coincidence rate and kappa of physician-collected specimens (endocervical), and vaginal self-collected samples assayed for HR-HPV by AmpFire and Cobas (95% CIs), and the associated histologic discrepancies

## Discussion

The CHIMUST dataset is highly validated due to liberal patient referral to colposcopy, and a minimum of 5 biopsies obtained from all colposcopy patients (POI protocol of directed and random biopsies) [1]. The AmpFire assay, for both self and direct collected samples, generated similar numbers of positive results and had similar accuracy compared with Cobas and SeqHPV with reference to histologic CIN2 or CIN3. Sensitivity was the same, while AmpFire was equal or more specific than COBAS but generally less specific than SeqHPV.

The comparable accuracy of AmpFire, in our opinion, tells only part of the story. Importantly, AmpFire does not require the complexities and costs of DNA extraction. A loss of viral copies, which can average about 40% with extraction, is reduced to a minimum by eliminating that step [14]. Dry brush samples can now be easily self-collected and delivered to simple non-specialized table-top laboratories [15]. Procedures are easy to learn because only basic pipetting skills are required. Product is not wasted since specimens can be processed individually, in small numbers, or batched.

The same equipment and methodology also being directly applicable for all routine sexually transmitted disease testing, and for SARS-CoV-2. Finally, this assay can function very effectively in a hospital laboratory or large clinic, but can be moved to less developed settings too.

In the future, the AmpFire assay might be redesigned to be a 14-type or even 13-type assay (removing type 53 and possibly type 66), although we had limited data to address the change. There were only 10 cases in our study cohort that were single type HPV 53 by AmpFire assay whom we discovered had colposcopy during the trial (5 were positive for other types by COBAS and/or SeqHPV), and all 10 had negative colposcopy and cytology <LSIL.

## Conclusion

We believe the data presented in this study demonstrates the AmpFire assay compares favorably with the Cobas and the SeqHPV assays. In consideration of the aforementioned characteristics, this assay may be a useful choice for primary screening and triage in low- and middle-income regions of the world, especially applicable to self-collection.

## Abbreviations

PCR: polymerase chain reaction
CHIMUST: Chinese multi-center screening tria
HPV: human papillomavirus
ECC: endocervical curettage
CIN: cervical intraepithelial neoplasia
CIN1: cervical intraepithelial neoplasia grade 1
CIN2: cervical intraepithelial neoplasia grade 2
CIN3: cervical intraepithelial neoplasia grade 3
AIS: Adenocarcinoma in situ
DNA: deoxyribonucleic acid
IRB: Institutional Review Board
POI: Preventive Oncology International, Inc.
FFPE: Formalin fixed paraffin embedded
CFDA: Chinese Food and Drug Administration
ASCUS: atypical squamous cells of undetermined significance
LSIL: low-grade grade squamous intraepithelial lesions
HSIL: high-grade squamous intraepithelial lesions
ASC-H: atypical squamous cells cannot rule out HSIL
